# Gender Differences in the Prevalence of Complementary and Alternative Medicine Utilization among Adults with Arthritis in the United States

**DOI:** 10.1155/2019/8739170

**Published:** 2019-11-03

**Authors:** Monira Alwhaibi, Yazed AlRuthia, Abdulkarim M. Meraya

**Affiliations:** ^1^Department of Clinical Pharmacy, College of Pharmacy, King Saud University, Riyadh, Saudi Arabia; ^2^Medication Safety Research Chair, College of Pharmacy, King Saud University, Riyadh, Saudi Arabia; ^3^Pharmacoeconomics Research Unit, College of Pharmacy, King Saud University, Riyadh, Saudi Arabia; ^4^Department of Clinical Pharmacy, College of Pharmacy, Jazan University, Jazan, Saudi Arabia; ^5^Pharmacy Practice Research Unit, College of Pharmacy, Jazan University, Jazan, Saudi Arabia

## Abstract

**Objective:**

To examine the association between sex and the use of complementary and alternative medicine (CAM) among adults with arthritis.

**Methods:**

Data from the 2012 National Health Interview Survey on CAM use for adults with arthritis were analyzed. Five different multivariable regression models were used to examine the association between sex and CAM use after adjusting for demographics, socioeconomics, perceived health status, functional limitations, comorbid chronic conditions, body mass index, and personal health practices.

**Results:**

The number of subjects who met the eligibility criteria and were eventually included in the study was 7,919 adults with arthritis. Around half of the study sample reported ever using CAM (*n* = 4,055), and about 27% (*n* = 2,016) reported using CAM in the past 12 months. Women have a significantly higher rate of ever utilization of CAM compared to their male counterparts (62.2% vs. 37.8%) as well as CAM use over the past 12 months (66.1% vs. 33.9%). After controlling for other covariates that can potentially affect the use of CAM, women had higher odds of ever using CAM (AOR = 1.68, 95% CI = 1.55–1.81) as well as the CAM use in the past 12 months (AOR = 1.63, 95% CI = 1.49–1.78) compared to men. Functional limitation and multiple comorbidities were associated with CAM use among women.

**Conclusions:**

The utilization rate of CAM among women with arthritis is significantly higher compared to their male counterparts, which highlights the need to screen adults with arthritis, particularly women, for potential drug-CAM interactions. Also, practicing patient-centered care is important, which should allow the patients to discuss the potential benefits and risks of CAM use with their healthcare providers.

## 1. Introduction

Arthritis is a highly prevalent chronic health condition in the United States (US), which is projected to reach 25% of the US adult population by 2030 [1]. According to the Institute for Health Metrics and Evaluation Global Burden of Disease project, arthritis is ranked as one of the highest contributors to global disability [2]. Furthermore, arthritis was associated with pain, poor health-related quality of life, and productivity loss based on multiple studies [3, 4]. Therefore, arthritis patients search for different therapies including complementary and alternative medicine (CAM) to alleviate pain and improve their quality of life. CAM therapies are diverse and include practices or products that are not part of conventional medicine [5]. CAM has been accepted and practiced worldwide, including in the US [6, 7].

According to a study that was conducted using nationally representative data of the US population to explore the prevalence of CAM utilization among patients with chronic health conditions, arthritis patients were found to be the highest users of CAM in comparison to other chronic health conditions [8]. It is estimated that around 30–41% of adults with arthritis in the US are CAM users [9–12]. Various modalities of CAM are commonly used for the treatment of arthritis such as homeopathy, acupuncture, naturopathy, chiropractic or osteopathic manipulation, and massage [12–17]. The main predicting factors for CAM use among adults with arthritis were believed to be the lack of effectiveness of conventional therapy [18], joint pain, and poor functional status [12]. However, it is notable that women with arthritis are using CAM more commonly than their male counterparts [9, 11]. In a cross-sectional study of adult patients with arthritis using the 2007 National Health Interview Survey (NHIS) data to investigate CAM use, a significant association between CAM use and gender was observed with women reporting higher utilization rate of CAM than their men's counterparts [11]. This was confirmed recently in the Zhang et al.'s studies using the 2012 NHIS data, where higher use of CAM among women was also noted [9, 10]. The higher utilization rate of CAM among women is believed to be due to its perceived benefits in improving the physical and mental wellbeing [10].

However, the extent to which women are using CAM more than their male counterparts among adults with arthritis needs to be explored further in order to understand the specific healthcare needs of each gender. Besides, the factors that influence the utilization of CAM among men and women with arthritis have been examined in a few studies with limited generalizability. Moreover, it is unknown which forms of CAM are most frequently used by women compared to men with arthritis. Thus, we aimed to address this research gap by exploring the extent of CAM utilization and potential factors that influence that utilization among men and women using a nationally representative sample of US adults with arthritis.

## 2. Methods

### 2.1. Data Source

The 2012 National Health Interview Survey (NHIS) data were used. The NHIS is an annual cross-sectional household interview survey of the noninstitutionalized US adult population. The sampling plan for the 2012 survey follows a multistage probability design permitting a representative sampling of households and noninstitutionalized population. Participants were randomly selected from each identified household [19]. The 2012 NHIS contains the following files: core files (household, family, person, and sample adult) and adult alternative medicine file [19]. The NHIS provide information on demographics, socioeconomic status, functional status, health status, chronic health conditions, and other variables. Chronic health conditions were identified by asking the participants, whether they have ever been told by a doctor or other health professionals that they had a chronic condition. Those who answered “yes” to having chronic condition(s) are then asked the following survey question: “Have you ever been told by a doctor or other health professional that you have some form of arthritis, rheumatoid arthritis, gout, lupus, or fibromyalgia?” [20]. The adult alternative medicine file was used to identify whether the respondent used CAM and the types of CAM used.

### 2.2. Study Sample

**Figure 1:**
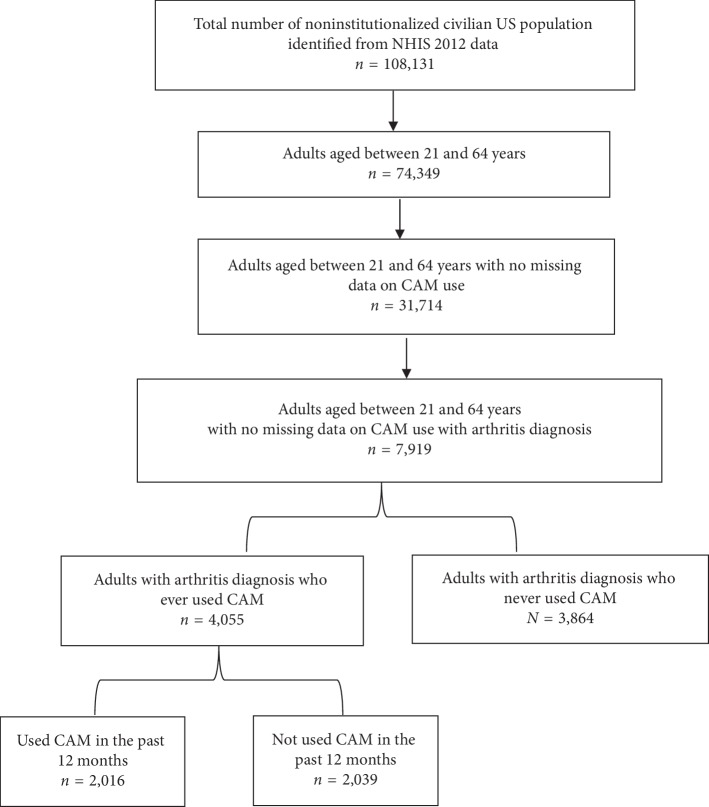
Flow diagram of study sample.

The study sample comprised adults aged >21 years with arthritis. Adults with missing data on CAM use variables were excluded.  Figure 1 displays the flow diagram of study sample.

### 2.3. Measures

#### 2.3.1. Dependent Variable

*CAM Use*. CAM users were categorized into binary variable: (1) have ever used any type of CAM; (2) have never used any type of CAM. The CAM users in the past 12 months were categorized into binary variable: (1) have used any CAM types in the past 12 months; (2) have not used any CAM types in the past 12 months. The reported types of CAM used were classified into three broad categories: (1) alternative medical systems (AMS), which included homeopathy, acupuncture, naturopathy, and Ayurveda; (2) manipulative and body-based therapies (MBBT) which included chiropractic or osteopathic manipulation, massage, Feldenkrais, Alexander technique, Trager psychophysical integration, craniosacral therapy, and Pilates; and (3) mind-body therapies (MBT) which included biofeedback, hypnosis, yoga, tai chi, and qi gong.

#### 2.3.2. Independent Variables

Demographics are composed of sex, age groups in years, race/ethnicity, and the region of residence. Socioeconomic status included education level, marital status, health insurance coverage, and poverty status. Other factors included perceived general health status (excellent, very good, good, fair, and poor), functional limitations, number of comorbid chronic conditions (0, 1, ≥2), and personal health practices (smoking status, alcohol use, and exercise). Body mass index (BMI) was categorized into underweight (<18.5 kg/m^2^); normal weight (18.5–24.9 kg/m^2^); overweight (25.0–29.9 kg/m^2^); and obese (30.0–40.0 kg/m^2^) [21].

### 2.4. Statistical Analysis

Frequencies and percentages were used to describe the categorical variables (e.g., age, sex, and marital status). Bivariate analyses were used to examine the sex differences in baseline characteristics and CAM use. Multiple binary logistic regression models were used to examine the adjusted relationships between sex and CAM use in which independent variables were entered in blocks. Adjusted odds ratios (AORs) and their corresponding 95% confidence intervals were used to present the results. Two-sided tests were used in all the statistical analyses, and a *p* value ≤ 0.05 was considered statistically significant. Model I assessed the association between sex and CAM use without adjusting for any independent variables (i.e., confounders). Model II adjusted for demographics (i.e., age, race/ethnicity, and the region of residence). Model III adjusted for socioeconomic status (i.e., education level, marital status, health coverage, and poverty status). Model IV adjusted for perceived health, functional limitations, and the number of chronic conditions. Lastly, model V adjusted for body mass index and personal health practices (smoking status, alcohol use, and exercise). In order to explore factors associated with CAM use among women and men, separate stratified (i.e., subgroup) binary logistic regression analyses among women and men were conducted. The sample adult weight (WTFA_SA) provided in the CAM module was used to account for the US population and missing observations [22]. The analyses controlled for the complex survey design of NHIS using SURVEYFREQ and PROC SURVEYLOGISTIC commands with strata (strat_p), cluster (psu_p), and weight (wtfa_sa) to determine weighted percentages and weighted regression. For the regression analyses, the chi-square goodness-of-fit test was used to assess the model fit. All analyses were performed using Statistical Analysis System Software (SAS 9.4 Institute Inc., Cary, NC, USA).

## 3. Results

### 3.1. Description of the Study Sample

Table 1 displays the characteristics of adults with arthritis study sample (*N* = 7,919) and the characteristics of adults with arthritis by sex. There were statistically significant differences between men and women in sociodemographic characteristics, functional limitations, number of comorbid chronic health conditions, body mass index, smoking status, alcohol use, and exercise. For example, a significantly higher percentage of women were poor (14.2% vs. 10.2%, *p* value < 0.001) and had functional limitation (62.4% vs. 37.6%, *p* value < 0.001) as compared to men.

### 3.2. CAM Use in the Study Sample

Around half of the study sample reported ever using CAM in general; however, only 26.7% of the study sample reported using CAM in the past 12 months (Table 2). The MBBT was reported to be the most commonly used CAM (20.8%) followed by MBT (8.9%) and AMS (5.6%) in the past 12 months. Chiropractic manipulation and osteopathic manipulation were most commonly used by the study sample (12%) followed by massage (11.6%) and yoga practice (7.2%).

### 3.3. Sex Differences in CAM Use

Table 2 shows the rate of CAM utilization by both men and women with arthritis across different variables. As compared to men, a significantly higher percentage of women reported using CAM at least once (66.2% vs. 37.8%), and 66.1% of women compared to 33.9% of men reported using CAM in the past 12 months. Even among the different types of CAM used in the past 12 months (AMS, MBBT, and MBT), women had a significantly higher percentage of use compared to men. Also, a significantly higher percentage of women were using homeopathy, acupuncture, naturopathy, massage, Pilates, biofeedback, and yoga as compared to men.

### 3.4. Sex Differences in CAM Use from Adjusted Analyses

Adjusted odds ratios (AORs) and 95% confidence intervals (CIs) on CAM use are displayed in Table 3. The odds of ever using CAM are significantly higher among women compared to men (OR = 1.24, 95% CI: 1.11–1.40). Also, the odds of using CAM in the past 12 months are significantly higher among women compared to men (OR = 1.40, 95% CI: 1.19–1.63) as shown in model I. This relationship remained significant even after controlling for a myriad of covariates as shown in models II–V (Table 3).

### 3.5. Sex Differences in Factors Affecting CAM Use

Men and women with functional limitations have higher odds to ever use CAM compared to those without functional limitation (AOR = 1.38, 95% CI = 1.09, 1.75 for women) (AOR = 1.39, 95% CI = 1.12, 1.73 for men) (Table 4). The number of chronic conditions was associated with ever using CAM among women but not men (AOR = 1.76, 95% CI = 1.33–2.33). Married men, but not women, had significantly lower odds of ever using CAM compared to their never married counterparts (AOR = 0.51, 95% CI = 0.37–0.71). Women, but not men, from the Northeast and Midwest had significantly lower odds of ever using CAM compared to their counterparts from the West (AOR = 0.47, 95% CI = 0.36–0.62; and AOR = 0.68, 95% CI = 0.53–0.87, respectively). Middle-income women, but not men, had significantly lower odds of using CAM compared to their high-income counterparts (AOR = 0.69, 95% CI = 0.55–0.87). Currently smoking women, but not men, had significantly lower odds of ever using CAM compared to their never-smoker counterparts (AOR = 0.69, 95% CI = 0.55–0.87). Current and past drinking women, but not men, had higher odds of ever using CAM compared to their light/abstaining alcohol drinking women (AOR = 1.44, 95% CI = 1.16–1.79; AOR = 1.87, 95% CI = 1.49–2.34, respectively). Moreover, women but not men, who exercise on a monthly or yearly basis as well as those who are unable to exercise had significantly lower odds of ever using CAM compared to their counterparts who exercise on a weekly basis (AOR = 0.51, 95% CI = 0.40–0.65; and, AOR = 0.33, 95% CI = 0.21–0.52, respectively). Only women, but not men, from the South as well as those with low income, underweight, and exercise on a monthly or yearly basis or unable to exercise at all had significantly lower odds of using CAM in the past 12 months compared to their counterparts who are from the West, with high income, normal weight, and exercise on a weekly basis as shown in Table 5.

## 4. Discussion

This study evaluated CAM use among adults with arthritis and provided understanding about the sex differences in CAM use across different variables. Nearly one out of two adults with arthritis reported using CAM which is higher than the rate published from the 2002 NHIS data in which around 41% of adults with arthritis reported using CAM [12]. Wide forms of CAM modalities were used by adults with arthritis in this study, and the most commonly reported type was the manipulative and body-based therapies which include, but not limited to, chiropractic or osteopathic manipulation and massage therapies. The higher rate of these therapies could be due to that these therapies are often covered by health insurance. Subjects with arthritis may use these types of therapies to reduce chronic pain and improve the functional status that accompanies arthritis.

The present study revealed that CAM use was more likely among women compared to men. Female patients with arthritis had higher odds of using CAM compared to their male counterparts despite controlling for a myriad of covariates in all of the performed statistical models, which highlights the strength of female gender in predicting higher utilization rate of CAM regardless of their demographic characteristics, socioeconomic characteristics, functional limitations, and medical characteristics. This finding is consistent with the literature among adults with arthritis [12] as well as adults in the general population [23–25]. A narrative review of 110 published studies has revealed that women had consistently higher rate of CAM use in community-based nonclinical population [24]. The higher utilization rate of CAM among women compared to men with arthritis could be attributable to their variable behavioral tendencies toward seeking any form of healthcare services [26] including visiting CAM providers; this tendency might be amplified when women suffer from arthritis. A study among the general US population reported higher utilization rate of CAM among females compared to their male counterparts mainly due to their positive perceptions about CAM and its impact on health and wellbeing [10].

This study has identified several factors that are associated with CAM use. Subgroup analyses revealed that poverty status, as well as education level, were significant factors for CAM use among men and women and adults with arthritis who were poor or had a lower education level were less likely to use CAM. This is consistent with other published studies among adults with arthritis as well as adults in the general population [10, 23, 24, 27]. Further, women who have two or more coexisting chronic conditions were more likely to use CAM. A review of health factors associated with CAM use revealed that CAM users tend to have more than one medical condition [24]. Besides, the functional limitation was a significant factor that affects CAM use among both men and women. In fact, poor functional status is one of the factors that influenced patients with arthritis decision of using CAM therapy [12]. Moreover, this study highlighted differences in the utilization of CAM among women themselves based on their ethnicity, weight, geographic location, and marital status, something that deserves further research to understand the main factors that have resulted in these differences.

Previously published research has found that the integration of CAM modalities and conventional treatments helps to improve the overall health of adults with arthritis [28, 29]. However, there is still inconsistency regarding the clinical efficacy and insufficient data about the safety of CAM modalities for arthritis due to the lack of well-designed clinical trials [13–16]. For instance, a systematic review of forty-three studies has evaluated the safety and efficacy of acupuncture for arthritis. The investigators concluded that acupuncture is beneficial to be used in rheumatoid arthritis to improve function and quality of life; however, there is still contradiction evidence for its clinical efficacy [29]. Therefore, adults with arthritis should use alternative therapies with caution. Besides, healthcare providers should be aware of the common CAM modalities, and also, they should discuss the possible benefits and harms of CAM use with their patients. In addition, different educational interventions customized based on the patients' response and needs should be created to improve patients' awareness of potential drug-CAM interactions that can render the antiarthritis drugs ineffective [30]. The possibility of adverse drug-CAM interactions is noteworthy especially when we know that approximately two-thirds of participants in this study had two or more chronic medical conditions, which increase their likelihood to use multiple medications leading to a higher risk of adverse drug-CAM interactions. This was reported in a study that assessed the prevalence of CAM use among adult patients with arthritis in Lebanon and found that 23% of the surveyed patients used CAM in addition to their prescription medications and around 24% sought medical care owing to potential drug-CAM side effects [31].

This study contributes to the wide literature on the use of CAM among adults with arthritis and includes a wide range of CAM modalities. It has also evaluated the gender disparities in CAM use after controlling for a comprehensive list of factors that affect CAM use. Findings of this study can be generalized to the US population. Further research should investigate whether the higher rate of CAM uses among women is due to inadequate access to healthcare, failure of women to adhere to their conventional treatments, or the inability of conventional medicine to adequately relieve arthritis. This study has some limitations. All measures were self-reported and therefore subject to recall bias. Other confounders such as the severity of arthritis-related pain, attitudes, and beliefs towards CAM use were not controlled for in the analysis because they were not assessed by the NHIS survey.

## 5. Conclusions

The utilization rate of CAM among women with arthritis is significantly higher compared to their male counterparts, which highlights the need to screen women with arthritis in particular for potential drug-CAM interactions. Also, the findings suggested that rheumatologists and other healthcare providers should be familiar of the most commonly used CAM modalities among women with arthritis and should discuss the possible benefits and harms of CAM use with their patients.

## Figures and Tables

**Table 1:** Description of the study sample by sex among adults with arthritis.

	Total sample		Women		Men		*p* value
	*N*	Wt %	*N*	Wt %	*N*	Wt %	
All	7,919	100.0	5,002	59.7	2,917	40.3	
Age in years							0.460
22–39 years	658	9.2	417	58.9	241	41.1	
40–49 years	942	13.6	585	58.6	357	41.4	
50–64 years	2,909	38.6	1,791	58.9	1,118	41.1	
65 and older	3,410	38.6	2,209	61.2	1,201	38.8	
Race/ethnicity							0.048
White	5,515	77.4	3,423	59.1	2,092	40.9	
African American	1,285	11.1	858	61.9	427	38.1	
Latino	783	8.1	518	64.4	265	35.6	
Other race	336	3.4	203	55.7	133	44.3	
Region							0.521
Northeast	1,332	17.6	859	61.0	473	39.0	
Midwest	1,758	24.9	1,120	60.8	638	39.2	
South	3,004	37.3	1,919	59.2	1,085	40.8	
West	1,825	20.2	1,104	58.2	721	41.8	
Marital status							<0.001
Married	3,739	63.2	2,024	53.2	1,715	46.8	
Widow/sep/div	3,296	28.8	2,419	73.9	877	26.1	
Never married	869	8.0	550	60.1	319	39.9	
Education level							0.295
LT high school	1,452	15.3	935	62.3	517	37.7	
High school	2,291	29.6	1,428	59.4	863	40.6	
GT high school	4,150	55.1	2,623	59.2	1,527	40.8	
Poverty status^†^							<0.001
Poor	1,293	11.8	934	68.1	359	31.9	
Near poor	1,565	16.4	1,026	63.0	539	37.0	
Middle income	2,029	26.7	1,238	58.8	791	41.2	
High income	2,107	32.9	1,186	54.2	921	45.8	
Missing	925	12.1	618	64.3	307	35.7	
Insurance							0.483
Insured	7,211	91.5	4,576	59.9	2,635	40.1	
Uninsured	690	8.5	414	58.2	276	41.8	
General health							0.186
Excellent	821	11.3	507	55.8	314	44.2	
Very good	1,991	27.2	1,243	59.7	748	40.3	
Good	2,655	33.6	1,676	59.8	979	40.2	
Fair	1,750	19.7	1,106	60.0	644	40.0	
Poor	700	8.2	469	64.0	231	36.0	
Functional limitation							<0.001
Yes	5,896	72.3	3,856	62.4	2,040	37.6	
No	2,017	27.7	1,143	52.6	874	47.4	
^#^Comorbid conditions							0.618
0	1,036	14.2	626	57.9	410	42.1	
1	1,602	21.2	1,007	60.4	595	39.6	
>=2	5,281	64.5	3,369	59.9	1,912	40.1	
Body mass index							<0.001
Underweight	113	1.2	97	87.5	16	12.5	
Normal weight	1,929	23.3	1,312	66.8	617	33.2	
Overweight	2,628	33.8	1,465	51.7	1,163	48.3	
Obese	2,994	38.0	1,893	58.4	1,101	41.6	
Missing	255	3.7	235	93.5	20	6.5	
Smoking status							<0.001
Never smoke	3,850	48.2	2,743	67.5	1,107	32.5	
Past smoker	2,587	34.0	1,377	50.0	1,210	50.0	
Current smoker	1,471	17.8	874	56.9	597	43.1	
Alcohol drinking							<0.001
Light/abstainer	1,587	17.4	1,290	79.6	297	20.4	
Former drinker	3,141	39.4	2,037	62.5	1,104	37.5	
Current drinker	3,134	42.7	1,640	49.2	1,494	50.8	
Missing	57	0.6	35	50.1	22	49.9	
Exercise							<0.001
Daily	427	5.3	230	49.0	197	51.0	
Weekly	1,677	23.3	941	52.2	736	47.8	
Monthly/yearly	5,350	65.8	3,509	62.9	1,841	37.1	
Unable	418	4.8	296	65.5	122	34.5	
Missing	47	0.7	26	52.1	21	47.9	

CAM use was based on adults, age over 21 years, who had arthritis. *p* values represent significant sex differences in baseline characteristics based on chi-square tests. Missing indicators for alcohol use, exercise, body mass index, and poverty status were presented in the table. Missing data for martial status (*n* = 15); education level (*n* = 26); insurance (*n* = 18); perceived health (*n* = 2); functional limitations (*n* = 6); and smoking (*n* = 11). ^†^Poor (<100% federal poverty line); near poor (100% to <200%); middle income (200% to <400%); and high income (≥400%). GT: greater than; LT: less than; Wt: weighted; #: number of chronic conditions; widow/sep/div: widowed, separated, and divorced.

**Table 2:** Number and weighted percent of complementary and alternative medicine use by sex among adults with arthritis.

	Total sample		Female		Male		*p* value
	*N*	Wt %	*N*	Wt %	*N*	Wt %	
All	7,919	100	5,002	59.7	2,917	40.3	
Any CAM use							<0.001
Any CAM ever	4,055	53.6	2,650	62.2	1,405	37.8	
No CAM ever	3,864	46.4	2,352	56.8	1,512	43.2	
CAM use past 12 months							<0.001
CAM past 12 months	2,016	26.7	1,390	66.1	626	33.9	
No CAM past 12 months	2,039	26.9	1,260	58.4	779	41.6	
No CAM ever	3,864	46.4	2,352	56.8	1,512	43.2	
Alternative medical system							0.002
AMS past 12 months	451	5.6	337	71.7	114	28.3	
No AMS past 12 months	7,446	94.4	4,648	59	2,798	41	
Manipulative and body-based							0.003
MBBT past 12 months	1,541	20.8	1,039	64.1	502	35.9	
No MBBT past 12 months	6,362	79.2	3,952	58.6	2,410	41.4	
Mind-body therapy							<0.001
MBT past 12 months	670	8.9	521	77.5	149	22.5	
No MBT past 12 months	7,224	91.1	4,465	58	2,759	42	
Type of CAM used past 12 months							
Homeopathy	199	2.7	154	71.7	45	28.3	0.016
Acupuncture	209	2.5	154	70.1	55	29.9	0.011
Naturopathy	82	1	63	78.7	19	21.3	0.004
Craniosacral	41	0.4	34	86	7	14	9.95
Ayurveda	23	0.3	19	83.2	4	16.8	0.049
Chiropractic/osteopathic	925	12.1	601	60.9	324	39.1	0.552
Massage	864	11.6	609	67.9	255	32.1	<0.001
Feldenkrais	5	0	3	71.8	2	28.2	0.652
Alexander Tech	9	0.1	4	51	5	49	0.636
Trager psychophysical	3	0	2	91.7	1	8.3	0.073
Pilates	116	1.5	97	86.5	19	13.5	<0.001
Biofeedback	48	0.8	36	79.7	12	20.3	0.014
Hypnosis	26	0.4	20	65.9	6	34.1	0.658
Yoga practice	532	7.2	429	81	103	19	<0.001
Tai chi	142	1.7	98	67.9	44	32.1	0.177
Qi gong	45	0.5	31	68.4	14	31.6	0.34

CAM use was based on 7,919 adults, age over 21 years, who had arthritis, and CAM use in the past 12 months was based on 4,055 CAM users. *p* values represent significant sex differences by complementary alternative medicine use based on chi-square tests. AMS: alternative medical system; CAM: complementary alternative medicine; MBBT: manipulative and body-based therapies; MBT: mind-body therapy; Wt: weighted.

**Table 3:** Adjusted odds ratios and 95% confidence intervals of women among adults with arthritis from logistic regressions on CAM use and CAM use in the past 12 months according to 2012 National Health Interview Survey.

	Ever used CAM (*N* = 4,055)			CAM use past 12 months (*N* = 3,864)		
	AOR	95% CI	*p* value	AOR	95% CI	*p* value
	Ref = (nonusers of CAM)			Ref = (nonusers of CAM in past 12 months)		
Model I, unadjusted						
Women	1.24	[1.11, 1.40]	<0.001	1.40	[1.19, 1.63]	<0.001
Men (ref)						

Model II, adjusted for demographics						
Women	1.30	[1.16, 1.46]	<0.001	1.41	[1.21, 1.66]	<0.001
Men (ref)						

Model III, adjusted for demographics and socioeconomics						
Women	1.38	[1.22, 1.57]	<0.001	1.47	[1.25, 1.73]	<0.001
Men (ref)						

Model IV, adjusted for demographics, socioeconomics, perceived general health status, functional limitations, number of comorbid chronic health conditions						
Women	1.37	[1.20, 1.55]	<0.001	1.47	[1.25, 1.73]	<0.001
Men (ref)						

Model V, adjusted for demographics, socioeconomics, perceived general health status, functional limitations, number of comorbid chronic health conditions, body mass index, smoking status, alcohol use, exercise.						
Women	1.54	[1.35, 1.75]	<0.001	1.50	[1.26, 1.78]	<0.001
Men (ref)						

Logistic regression on CAM use was based on adults, age over 21 years, who had arthritis. Logistic regression on CAM use in the past 12 months was based on 4,055 CAM users. *p* values represent significant sex differences based on logistic regressions on CAM use and CAM use in the past 12 months after controlling for demographic characteristics, socioeconomic status, perceived general health status, functional limitations, number of comorbid chronic health conditions, body mass index, smoking status, alcohol use, and exercise. AOR: adjusted odds ratios; CI: confidence interval; CAM: complementary alternative medicine; ref: reference group.

**Table 4:** Factors affecting ever used CAM for men and women adjusted odds ratios and 95% confidence intervals from logistic regressions according to 2012 National Health Interview Survey.

	Women			Men		
	AOR	95% CI	*p* value	AOR	95% CI	*p* value
Age group						
22–39 (Ref.)						
40–49	0.94	[0.68, 1.31]	0.727	1.01	[0.67, 1.53]	0.966
50–64	0.81	[0.61, 1.07]	0.132	0.80	[0.56, 1.13]	0.208
65, +	0.51	[0.37, 0.71]	<0.0001	0.64	[0.43, 0.95]	0.025

Race/ethnicity						
White (Ref.)						
African American	0.50	[0.40, 0.62]	<0.0001	0.45	[0.33, 0.61]	<0.0001
Latino	0.70	[0.53, 0.92]	0.011	0.60	[0.42, 0.86]	0.006
Other	0.68	[0.44, 1.06]	0.088	0.60	[0.36, 1.00]	0.050

Marital status						
Never married (Ref.)						
Married	1.07	[0.81, 1.41]	0.615	1.43	[1.02, 2.00]	0.037
Widow/sep/div	1.08	[0.83, 1.40]	0.575	1.48	[1.06, 2.07]	0.022

Education level						
GT high school (Ref.)						
LT high school	0.41	[0.32, 0.52]	<0.0001	0.52	[0.37, 0.71]	<0.0001
High school	0.55	[0.45, 0.66]	<0.0001	0.72	[0.57, 0.90]	0.004

Region						
West (Ref.)						
Northeast	0.47	[0.36, 0.62]	<0.0001	0.76	[0.54, 1.08]	0.122
Midwest	0.68	[0.53, 0.87]	0.0024	0.77	[0.57, 1.04]	0.084
South	0.47	[0.37, 0.61]	<0.0001	0.55	[0.42, 0.72]	<0.0001

Poverty status^†^						
High income (Ref.)						
Poor	0.49	[0.37, 0.66]	<0.0001	0.52	[0.33, 0.82]	0.005
Near poor	0.71	[0.55, 0.93]	0.012	0.63	[0.46, 0.87]	0.005
Middle income	0.69	[0.55, 0.87]	0.002	0.84	[0.65, 1.09]	0.194
Missing	0.78	[0.59, 1.04]	0.096	0.71	[0.51, 0.99]	0.045

Insurance						
Insured (Ref.)						
Uninsured	1.12	[0.83, 1.52]	0.453	1.42	[0.99, 2.05]	0.059

General health						
Excellent (Ref.)						
Very good	1.05	[0.76, 1.43]	0.776	1.03	[0.72, 1.46]	0.871
Good	1.04	[0.76, 1.44]	0.793	1.27	[0.90, 1.79]	0.175
Fair	1.01	[0.69, 1.47]	0.977	1.09	[0.75, 1.58]	0.664
Poor	0.91	[0.59, 1.39]	0.651	1.58	[0.96, 2.58]	0.069

Functional limitation						
No (Ref.)						
Yes	1.38	[1.09, 1.75]	0.008	1.39	[1.12, 1.73]	0.003

^#^Comorbid conditions						
0 (Ref.)						
1	1.21	[0.90, 1.63]	0.2139	1.06	[0.77, 1.44]	0.727
>=2	1.76	[1.33, 2.33]	<0.0001	0.96	[0.71, 1.28]	0.760

Body mass index						
Normal weight (Ref.)						
Under weight	1.21	[0.69, 2.11]	0.502	0.26	[0.05, 1.25]	0.093
Over weight	0.94	[0.75, 1.17]	0.554	0.86	[0.67, 1.10]	0.225
Obese	0.95	[0.77, 1.18]	0.661	0.85	[0.67, 1.09]	0.210
Missing	0.75	[0.49, 1.14]	0.176	1.53	[0.47, 4.96]	0.475

Smoking status						
Never smoke (Ref.)						
Past smoker	1.11	[0.90, 1.35]	0.329	1.18	[0.94, 1.47]	0.153
Current smoker	0.78	[0.63, 0.97]	0.026	0.83	[0.62, 1.12]	0.223

Alcohol drinking						
Light/abstainer (Ref.)						
Former drinker	1.44	[1.16, 1.79]	0.001	1.29	[0.92, 1.80]	0.142
Current drinker	1.87	[1.49, 2.34]	<0.0001	1.19	[0.83, 1.70]	0.343
Missing	0.72	[0.32, 1.63]	0.432	1.26	[0.43, 3.68]	0.673

Exercise						
Weekly (Ref.)						
Daily	1.07	[0.69, 1.65]	0.769	1.05	[0.70, 1.60]	0.806
Monthly/yearly	0.51	[0.40, 0.65]	<0.0001	0.90	[0.70, 1.16]	0.433
Unable	0.33	[0.21, 0.52]	<0.0001	0.81	[0.46, 1.41]	0.457
Missing	1.32	[0.45, 3.90]	0.612	0.32	[0.10, 1.06]	0.062

Logistic regression on CAM use by sex was based on adults, age over 21 years, who had arthritis. Logistic regression on ever used CAM use was based on 7, 919 observations. *p* values represent significant group differences based on logistic regressions on CAM use. ^†^Poor (<100% federal poverty line); near poor (100% to <200%); middle income (200% to <400%); and high income (≥400%). AOR: adjusted odds ratios; CI: confidence interval; CAM: complementary alternative medicine; Ref.: reference group; widow/sep/div: widowed, separated, and divorced.

**Table 5:** Factors affecting CAM use in the past 12 months for men and women adjusted odds ratios and 95% confidence intervals from logistic regressions according to 2012 National Health Interview Survey.

	Women			Men		
	AOR	95% CI	*p* value	AOR	95% CI	*p* value
Age group						
22–39 (Ref.)						
40–49	0.67	[0.44, 1.04]	0.074	0.62	[0.34, 1.14]	0.125
50–64	0.46	[0.32, 0.66]	<.0001	0.48	[0.27, 0.84]	0.011
65, +	0.34	[0.23, 0.51]	<.0001	0.40	[0.21, 0.74]	0.003

Race/ethnicity						
White (Ref.)						
African American	0.96	[0.67, 1.37]	0.818	1.16	[0.73, 1.83]	0.527
Latino	0.86	[0.60, 1.25]	0.430	1.14	[0.67, 1.96]	0.63
Other	1.69	[0.97, 2.94]	0.064	1.01	[0.57, 1.77]	0.973

Marital status						
Never married (Ref.)						
Married	0.93	[0.65, 1.32]	0.679	1.23	[0.68, 2.22]	0.489
Widow/sep/div	1.00	[0.69, 1.44]	0.982	0.88	[0.47, 1.65]	0.689

Education level						
GT high school (Ref.)						
LT high school	0.65	[0.44, 0.95]	0.026	0.85	[0.53, 1.38]	0.512
High school	0.84	[0.66, 1.07]	0.157	0.67	[0.47, 0.95]	0.025

Region						
West (Ref.)						
Northeast	1.17	[0.86, 1.60]	0.307	1.09	[0.74, 1.60]	0.675
Midwest	0.98	[0.73, 1.32]	0.904	0.95	[0.64, 1.40]	0.785
South	0.73	[0.56, 0.94]	0.014	1.02	[0.71, 1.47]	0.922

Poverty status^†^						
High income (Ref.)						
Poor	0.43	[0.29, 0.65]	<.0001	0.96	[0.51, 1.84]	0.909
Near poor	0.65	[0.46, 0.91]	0.012	0.95	[0.56, 1.61]	0.859
Middle income	0.65	[0.48, 0.89]	0.006	0.90	[0.64, 1.27]	0.553
Missing	0.56	[0.39, 0.81]	0.002	1.16	[0.69, 1.95]	0.570

Insurance						
Insured (Ref.)						
Uninsured	0.98	[0.67, 1.43]	0.923	0.91	[0.56, 1.48]	0.693

General health						
Excellent (Ref.)						
Very good	0.93	[0.64, 1.34]	0.686	1.10	[0.70, 1.74]	0.669
Good	0.87	[0.59, 1.27]	0.469	0.93	[0.58, 1.51]	0.774
Fair	0.83	[0.53, 1.30]	0.406	1.18	[0.68, 2.05]	0.558
Poor	0.92	[0.53, 1.57]	0.7538	0.69	[0.33, 1.41]	0.305

Functional limitation						
No (Ref.)						
Yes	0.78	[0.60, 1.01]	0.058	1.29	[0.93, 1.78]	0.129

# Comorbid conditions						
0 (Ref.)						
1	1.26	[0.87, 1.83]	0.227	0.98	[0.62, 1.53]	0.913
>=2	1.33	[0.92, 1.90]	0.125	0.73	[0.46, 1.15]	0.179

Body mass index						
Normal weight (Ref.)						
Underweight	0.37	[0.14, 0.94]	0.038	1.24	[0.16, 9.46]	0.837
Overweight	0.83	[0.63, 1.10]	0.193	0.97	[0.65, 1.45]	0.885
Obese	0.77	[0.59, 1.02]	0.072	0.92	[0.58, 1.44]	0.707
Missing	0.87	[0.50, 1.53]	0.634	1.43	[0.30, 6.74]	0.653

Smoking status						
Never smoke (Ref.)						
Past smoker	0.91	[0.72, 1.17]	0.474	0.97	[0.68, 1.39]	0.863
Current smoker	0.74	[0.55, 0.99]	0.043	0.58	[0.38, 0.88]	0.011

Alcohol drinking						
Light/abstainer (Ref.)						
Former drinker	1.15	[0.86, 1.56]	0.347	1.22	[0.72, 2.06]	0.454
Current drinker	1.16	[0.84, 1.61]	0.356	1.37	[0.80, 2.36]	0.249
Missing	0.51	[0.09, 2.72]	0.427	0.34	[0.05, 2.41]	0.280

Exercise						
Weekly (Ref.)						
Daily	0.83	[0.51, 1.37]	0.471	1.49	[0.88, 2.53]	0.136
Monthly/yearly	0.69	[0.52, 0.93]	0.015	0.74	[0.52, 1.07]	0.107
Unable	0.53	[0.28, 0.99]	0.047	0.89	[0.39, 2.04]	0.785
Missing	0.59	[0.15, 2.36]	0.457	0.69	[0.14, 3.55]	0.662

Logistic regression on CAM use by sex was based on adults, age over 21 years, who had arthritis. Logistic regression on CAM use in the past 12 months was based on 4, 055 CAM users. *p* values represent significant group differences based on logistic regressions on CAM use in the past 12 months. ^†^Poor ((<100% federal poverty line); near poor (100% to <200%); middle income (200% to <400%); and high income (>400%)). AOR: adjusted odds ratios; CI: confidence Interval; CAM: complementary alternative medicine; Ref.: reference group.

## Data Availability

The data used to support the findings of this study are available from the 2012 NHIS and openly made available from the National Center for Health Statistics (NCHS) or the Centers for Disease Control and Prevention (CDC) at https://www.cdc.gov/nchs/nhis/nhis_2012_data_release.htm.

## Abbreviations

CAM: Complementary and alternative medicine
US: United States
NHIS: National Health Interview Survey
AMS: Alternative medical systems
MBBT: Manipulative and body-based therapies
MBT: Mind-body therapies
BMI: Body mass index
AORS: Adjusted odds ratios
CI: Confidence intervals.
